# Impact of timing to initiate adjuvant therapy on survival of elderly glioblastoma patients using the SEER-Medicare and national cancer databases

**DOI:** 10.1038/s41598-023-30017-z

**Published:** 2023-02-25

**Authors:** Ping Zhu, Xianglin L. Du, Lu-yu Hwang, David Lairson, Ruosha Li, Yoshua Esquenazi, Jay-Jiguang Zhu

**Affiliations:** 1 The Vivian L. Smith Department of Neurosurgery, The University of Texas Health Science Center at Houston McGovern Medical School, Houston, TX USA; 2 Epidemiology, Human Genetics and Environmental Sciences, UTHealth School of Public Health, Houston, TX USA; 3 Department of Management, Policy, and Community Health, School of Public Health, The University of Texas Health Science Center at Houston, Houston, TX USA; 4 Department of Biostatistics and Data Science, The University of Texas Health Science Center at Houston, Houston, TX USA; 5 Center for Precision Health, School of Biomedical Informatics, The University of Texas Health Science Center at Houston, Houston, TX USA; 6 The Vivian L. Smith Department of Neurosurgery, The University of Texas Health Science Center at Houston (UTHealth) McGovern Medical School, 6400 Fannin Street, Suite 2800, Houston, TX 77030 USA

**Keywords:** Oncology, Epidemiology

## Abstract

The optimal time to initiate adjuvant therapy (AT) in elderly patients with glioblastoma (GBM) remains unclear. We investigated the impact of timing to start AT on overall survival (OS) using two national-scale datasets covering elderly GBM populations in the United States. A total of 3159 and 8161 eligible elderly GBM patients were derived from the Surveillance, Epidemiology and End Results (SEER)—Medicare linked dataset (2004–2013) and the National Cancer Database (NCDB) (2004–2014), respectively. The intervals in days from the diagnosis to the initiation of AT were categorized based on two scenarios: Scenario I (quartiles), ≤ 15, 16–26, 27–37, and ≥ 38 days; Scenario II (median), < 27, and ≥ 27 days. The primary outcome was OS. We performed the Kaplan–Meier and Cox proportional hazards regression methods for survival analysis. A sensitivity analysis was performed using Propensity Score Matching (PSM) method to achieve well-balanced characteristics between early-timing and delayed-timing in Scenario II. Improved OS was observed among patients who underwent resection and initiated AT with either a modest delay (27–37 days) or a longer delay (≥ 38 days) compared to those who received AT immediately (≤ 15 days) from both the SEER-Medicare dataset [adjusted hazard ratio (aHR) 0.74, 95% CI 0.64–0.84, *P* < 0.001; and aHR 0.81, 95% CI 0.71–0.92, *P* = 0.002] and the NCDB (aHR 0.83, 95% CI 0.74–0.93, *P* = 0.001; and aHR 0.87, 95% CI 0.77–0.98, *P* = 0.017). The survival advantage is observed in delayed-timing group as well in Scenario II. For elderly patients who had biopsy only, improved OS was only detected in a longer delay (Scenario I: ≥ 38 days vs. ≤ 15 days) or the delayed-timing group (Scenario II: ≥ 27 days vs. < 27 days) in the NCDB while no survival difference was seen in SEER-Medicare population. For the best timing to start AT in elderly GBM patients, superior survivals were observed among those who had craniotomy and initiated AT with a modest (27–37 days) or longer delays (≥ 38 days) following diagnosis using both the SEER-Medicare and NCDB datasets (Scenario I). Such survival advantage was confirmed when categorizing delayed-timing vs. early-timing with the cut-off at 27 day in both datasets (Scenario II). The increased likelihood of receiving delayed AT (≥ 27 days) was significantly associated with tumor resection (STR/GTR), years of diagnosis after 2006, African American and Hispanics races, treatments at academic facilities, and being referred. There is no difference in timing of AT on survival among elderly GBM patients who had biopsy in the SEER-Medicare dataset. In conclusion, initiating AT with a modest delay (27–37 days) or a longer delay (≥ 38 days) after craniotomy may be the preferred timing in the elderly GBM population.

## Introduction

Glioblastoma (GBM) is the most common malignant primary brain tumor, accounting for approximately 48.6% of primary malignant brain tumors^1^. The median overall survival (OS) of GBM patients range from 14.6 to 20.9 months for randomized clinical trial (RCT) participants, 11 and 9.3 months in all GBM patients and elderly GBM patients in real world setting, respectively^2–5^.

The standard of care of newly diagnosed GBM is maximum safe resection followed by concurrent radiation and oral daily temozolomide (TMZ) chemotherapy and then adjuvant TMZ, 5 days on and 23 days off for 6–12 cycles^1,2^. Biopsy is offered to patients with tumors’ eloquent locations, significant comorbidity or frail health. Identification of the best timing to initiate adjuvant therapy (AT) has been considered as an important factor in aiding GBM control. Traditionally, treating physicians start AT soon after pathological diagnosis, surgical wound recovery and discharge from rehabilitation facility. Other factors delaying AT due to logistic reasons include visiting radiation oncologist and neuro-oncologist or medical oncologist as well insurance approval of radiation and chemotherapy plus dispensary of TMZ from pharmacy^6^. Further delay may occurs when patients need second opinions, transfer care to a tertiary medical center or participate clinical trials. Most patients started AT approximately 3–6 weeks after craniotomy and sooner for patient who had biopsy only (35 days^2^, within 5 weeks + 28 days^7^, 28 days after last RT^8^, 29–48 days^9^, 3.8 months^5^). Prior studies have investigated the association between timing of AT and GBM patients’ outcomes by retrospective data analyses only as there is no clinical trial performed addressing this question^10–13^. However, these retrospective studies generated controversial findings. Five studies demonstrated the association of delayed-timing of radiation (RT)/chemoradiation (CRT) with an improved GBM survival^10–14^, while one study reported an inferior outcome of long-delayed initiation of concurrent chemoradiation (CCRT) on GBM survival^15^. Three studies found no difference in GBM survival across different timings (Table S1)^16–18^. There is few study addressing this issue in elderly GBM patients only.

Several limitations existed in the aforementioned nine studies: (1) Only one study focused on elderly GBM patients^16^ and included GBM patients diagnosed from 1991 to 2002 only, which was prior to the era of Stupp protocol of concurrent RT with TMZ based on EORTC-NCIC trial^2^; (2) All of these studies applied different thresholds of timing groups and none of them validated their results in another comparable dataset; (3) Only three studies explored the predictors of delayed RT/CRT timing and could not reach a consistent conclusion^12,13,16^; (4) Four studies had limited sample sizes (N < 700) including RCT data, local hospital records, or TCGA^11,15,17,18^.

Therefore, the optimal AT timing and the related predictors of delayed AT remain uncertain for GBM patients, particularly the elderly GBM population. The objectives of the present study are to investigate the survival impact of AT timing as well as the related potential predictors using two large national cohorts of elderly GBM patients from the SEER-Medicare and NCDB datasets.

## Materials and methods

### Datasets and populations

This is a retrospective cohort study of elderly GBM patients derived from the SEER-Medicare (2004–2013) and NCDB datasets (2004–2014). The SEER-Medicare linked dataset collected cancer data across 18 population-based cancer registries, covering approximately 34% of the U.S. population. The Medicare program is a federally funded primary health insurance for approximately 97% elderly patients aged 65 years and older in the U.S. The NCDB is a nation-wide, hospital-based oncology database that collects surveillance cancer data as previously described^19^. The variable settings of core parameters in the NCDB are similar to those collected in the SEER. We chose the NCDB dataset from 2004 to 2014 to best match the data period collected in SEER-Medicare (2004–2013).

### Study subjects and study periods

De-identified newly diagnosed elderly GBM patients (age at diagnosis ≥ 65) from the SEER-Medicare between January 2004 and December 2013 and from the NCDB Participant User File (PUF) between January 2004 and December 2014 were queried. GBM was defined by the International Classification of Disease for Oncology, third edition (ICD-O-3) coded as 9440, 9441, or 9442^20^, with topography codes C710–C719^21,22^. Exclusion criteria are described in Supplementary Materials and Fig. S1.

### Predictors, covariates, and outcomes

#### Primary exposure variables

For the SEER-Medicare database, time to AT was calculated as days from the date of diagnosis to the date of AT initiation, and it was categorized based on two scenarios: Scenario I (quartiles, days): 0–15, 16–26, 27–37, and ≥ 38; Scenario II (median, days): < 27 (early-timing), and ≥ 27 (delayed-timing).For the NCDB database, we classified the timing of AT using the same thresholds of the SEER-Medicare, which is used as a validation dataset.

#### Covariates

For the SEER-Medicare dataset, covariates include socio-demographics, facility/SEER registry features, and clinical treatments. Surgery and AT were identified by MEDPAR (Medicare Provider Analysis and Review), NCH (National Claims History), outpatient, DME (Durable Medical Equipment), and Part D Event files in Medicare claims. The details of coding were presented in Supplemental Table S2. We included the Medicare claims from 1999 and successfully evaluated the comorbidity during one year of claims prior to diagnosis for all cases. This data was used to calculate pre-diagnostic Charlson Comorbidity Scores.

For the NCDB database, socio-demographics settings are similar to the SEER-Medicare data. Facility features included facility location, facility type, residence-hospital distance, and care transition. Charlson/Deyo comorbidity score was a weighted score derived from the sum of the scores for each of comorbid conditions and classified into 0, 1, and ≥ 2. Care transition/treatment referral occurred if a patient underwent treatment at multiple facilities. The utilization of adjuvant RT or chemotherapy was defined as yes or no. More details about the definition of covariates were presented in supplementary materials.

#### Outcomes

OS was measured as the time interval in months from diagnosis to death or last visit. Those who were still alive by the last date of follow-up (December 31, 2014) were censored. Additionally, we examined the predictors associated with improved survival related to delayed-timing (≥ 27 days) versus early-timing (< 27 days).

### Statistical analysis

The Mann–Whitney *U* tests and Pearson's χ^2^ tests were performed to compare patient characteristics by groups of AT timing. Univariable and multivariable binary logistic regression models were utilized to identify the predictors of delayed AT. The Hosmer–Lemeshow test was applied to check the goodness-of-fit of the regression models. OS was assessed by using Kaplan–Meier methods, and the difference across survival functions was tested by a two-sided log-rank test.

Multivariable Cox proportional hazards regression analyses were conducted to assess the impact of time to AT on survival by controlling covariates. Stratification analysis by surgery type (biopsy or resection) were also examined. The regression models rendered adjusted hazard ratio (aHR) estimates and 95% confidence interval (CI). A sensitivity analysis was performed using PSM method to achieve well-balanced characteristics between early-timing and delayed-timing (supplementary materials). Statistical analyses were performed with Stata IC 15.1 (StataCorp, College Station, TX). *P* values were two-sided and considered statistically significant at *P* < 0.05.

### Ethics statement

This study was approved by the Committee for the Protection of Human Subjects at the University of Texas Health Science Center at Houston. All methods were performed in accordance with the relevant guidelines and regulations. The need for informed consent was waived by Committee for the Protection of Human Subjects at the University of Texas Health Science Center at Houston due to retrospective nature of the study.

## Results

### SEER-Medicare dataset

A total of 3159 elderly GBM patients were included, with a median time to AT of 26 days (range 0–89 days). The median age at diagnosis was 73 years (IQR 69–78, range 65–95, years). The majority of patients were Caucasians (91.7%), married (69.1%), resided in metropolitan areas (82.3%), and treated at teaching hospitals (64.9%). In Table 1, the distributions of surgery (resection vs. biopsy), AT (non-CRT vs. CRT), and chemotherapy agents (None vs. TMZ vs. Other chemotherapy agents) varied significantly across times to start AT (All *P* < 0.001). Patients who underwent gross total resection (GTR) or subtotal resection (STR) were more likely to experience delayed-timing (≥ 27 days) of AT compared to those who underwent biopsy (Table S7).

**Table 1:** Patient characteristics in SEER-Medicare analytic cohort by time to AT (N = 3159).

Characteristics, N (%)	Time to AT				
	≤ 15 days	16–26 days	27–37 days	≥ 38 days	P*
Patients	805	830	766	758	
Socio-demographics					
Age at diagnosis, median (IQR)	73 (69, 78)	73 (69, 77)	72 (68, 77)	73 (69, 77)	0.021
Age at diagnosis, years					0.280
65–74	495 (61.5)	536 (64.6)	506 (66.1)	489 (64.5)	
75–90	310 (38.5)	294 (35.4)	260 (33.9)	269 (35.5)	
Gender					0.700
Male	425 (52.8)	458 (55.2)	409 (53.4)	398 (52.5)	
Female	380 (47.2)	372 (44.8)	357 (46.6)	360 (47.5)	
Year of diagnosis					0.150
1/2004–12/2005	208 (25.8)	240 (28.9)	236 (30.8)	204 (26.9)	
1/2006–12/2007	155 (19.3)	150 (18.1)	130 (17.0)	156 (20.6)	
1/2008–12/2009	147 (18.3)	152 (18.3)	142 (18.5)	169 (22.3)	
1/2010–12/2011	198 (24.6)	190 (22.9)	173 (22.6)	149 (19.7)	
1/2012–12/2013	97 (12.0)	98 (11.8)	85 (11.1)	80 (10.6)	
Race/ethnicity					0.440
White	743 (92.3)	761 (91.7)	711 (92.8)	682 (90.0)	
Black	28 (3.5)	31 (3.7)	22 (2.9)	29 (3.8)	
Hispanic	NA	14 (1.7)	NA	16 (2.1)	
Others	NA	24 (2.9)	NA	31 (4.1)	
Marital status					0.900
Single/DWS	240 (29.8)	258 (31.1)	240 (31.3)	237 (31.3)	
Married	565 (70.2)	572 (68.9)	526 (68.7)	521 (68.7)	
Residential location					0.910
Metropolitan	667 (82.9)	687 (82.8)	627 (81.9)	620 (81.8)	
Urban/rural	138 (17.1)	143 (17.2)	139 (18.1)	138 (18.2)	
Education, %					0.360
≥ 29	104 (12.9)	102 (12.3)	91 (11.9)	107 (14.1)	
20–28.9	122 (15.2)	132 (15.9)	126 (16.4)	135 (17.8)	
14–19.9	121 (15.0)	148 (17.8)	137 (17.9)	137 (18.1)	
< 14	458 (56.9)	448 (54.0)	412 (53.8)	379 (50.0)	
Income, dollars					0.620
< 30,000	105 (13.0)	96 (11.6)	90 (11.7)	103 (13.6)	
30,000–35,999	78 (9.7)	88 (10.6)	78 (10.2)	83 (10.9)	
36,000–45,999	159 (19.8)	153 (18.4)	154 (20.1)	165 (21.8)	
≥ 46,000	463 (57.5)	493 (59.4)	444 (58.0)	407 (53.7)	
Facility characteristics					
Registry location					0.640
Northeast	167 (20.7)	188 (22.7)	140 (18.3)	153 (20.2)	
North Central	118 (14.7)	107 (12.9)	114 (14.9)	109 (14.4)	
South	179 (22.2)	195 (23.5)	176 (23.0)	164 (21.6)	
West	341 (42.4)	340 (41.0)	336 (43.9)	332 (43.8)	
Teaching status					0.270
No	285 (35.4)	286 (34.5)	288 (37.6)	249 (32.8)	
Yes	520 (64.6)	544 (65.5)	478 (62.4)	509 (67.2)	
Clinical treatments					
Surgery					< 0.001
Biopsy	354 (44.0)	339 (40.8)	276 (36.0)	263 (34.7)	
Resection	451 (56.0)	491 (59.2)	490 (64.0)	495 (65.3)	
AT					< 0.001
Non-CRT	327 (40.6)	282 (34.0)	242 (31.6)	253 (33.4)	
CRT	478 (59.4)	548 (66.0)	524 (68.4)	505 (66.6)	
Chemotherapy agents					< 0.001
None	302 (37.5)	265 (31.9)	236 (30.8)	242 (31.9)	
TMZ	420 (52.2)	451 (54.3)	427 (55.7)	378 (49.9)	
Other chemotherapy agents	83 (10.3)	114 (13.7)	103 (13.4)	138 (18.2)	
Charlson comorbidity score					0.066
0	401 (49.8)	451 (54.3)	412 (53.8)	396 (52.2)	
1	207 (25.7)	196 (23.6)	212 (27.7)	206 (27.2)	
≥ 2	197 (24.5)	183 (22.0)	142 (18.5)	156 (20.6)	

*AT* adjuvant therapy, *DWS* divorced/widowed/separated, *RT* radiation therapy, *CRT* chemoradiation, *TMZ* temozolomide, *NA* not available, was presented once the number of patients in a specific cell was 11 or less based on privacy policy of SEER-Medicare.

*Pearson's χ^2^ test or Mann–Whitney *U* test were conducted to compare the proportions of baseline characteristics across time to AT.

**Figure 1:**
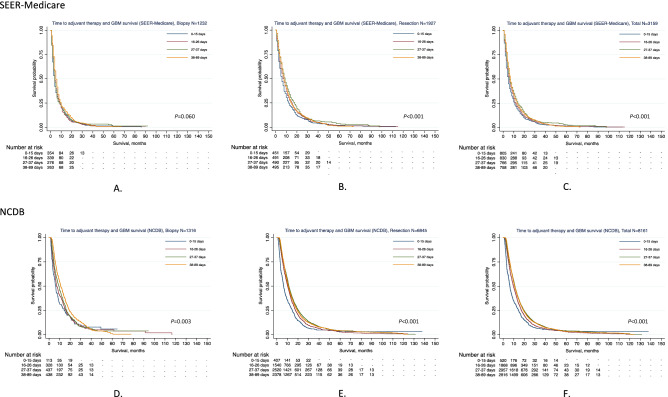
OS of GBM patients across time to AT (four-category) by applying the Kaplan–Meier method. *“−” was used when the number of patients was 11 or fewer based on privacy policy of both SEER-Medicare and NCDB. (**A**) OS of GBM across time to AT in patients undergoing biopsy from SEER-Medicare. (**B**) OS of GBM across time to AT in patients undergoing resection from SEER-Medicare. (**C**) OS of GBM across time to AT among total patients from SEER-Medicare. (**D**) OS of GBM across time to AT in patients undergoing biopsy from NCDB. (**E**) OS of GBM across time to AT in patients undergoing resection from NCDB. (**F**) OS of GBM across time to AT among total patients from NCDB.

**Figure 2:**
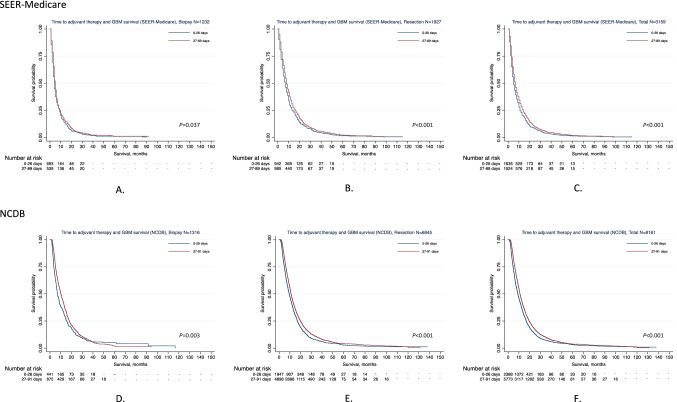
OS of GBM patients across time to AT (two-category) by applying the Kaplan–Meier method. *“−” was used once the number of patients was 11 or fewer based on privacy policy of both SEER-Medicare and NCDB. (**A**) OS of GBM across time to AT in patients undergoing biopsy from SEER-Medicare. (**B**) OS of GBM across time to AT in patients undergoing resection from SEER-Medicare. (**C**) OS of GBM across time to AT among total patients from SEER-Medicare. (**D**) OS of GBM across time to AT in patients undergoing biopsy from NCDB. (**E**) OS of GBM across time to AT in patients undergoing resection from NCDB. (**F**) OS of GBM across time to AT among total patients from NCDB.

The median OSs were 5, 6, 6, and 7 months across the 4 quartiles of AT timing for all patients in SEER-Medicare, respectively (*P* < 0.001) in Scenario I (Table 3). For the resection cohort, patients who started AT between 27–37 days experienced the longest median OS (9 months) compared to the remaining 3 groups (≤ 15 days: 6, 16–26 days: 7, ≥ 38 days: 8 months; *P* < 0.001). Similar results were observed in Scenario II (median OS, < 27 days vs. ≥ 27 days: 7 vs. 8 months; *P* < 0.001). The Kaplan–Meier plots of survival curves are presented in Fig. 1A–C (Scenario I) and Fig. 2A–C (Scenario II).

As shown in Table 4, in the resection cohort, patients who began AT between 27 and 37 days (aHR 0.74, 95% CI 0.64–0.84, *P* < 0.001) and ≥ 38 days (aHR 0.81, 95% CI 0.71–0.92, *P* = 0.002) experienced improved OS. This survival benefit is observed in delayed-timing group in Scenario II ( ≥ 27 days vs. < 27 days, aHR 0.82, 95% CI 0.74–0.90, *P* < 0.001). Similar findings were observed in the total cohort: Scenario I, aHR 0.86, 95% CI 0.78–0.96, *P* = 0.005 (27–37 days vs. ≤ 15 days), aHR 0.86, 95% CI 0.78–0.95, *P* = 0.004 (≥ 38 days vs. ≤ 15 days); Scenario II, aHR 0.88, 95% CI 0.82–0.94, *P* < 0.001 (≥ 27 days vs. < 27 days). No survival difference across timing groups was detected in the biopsy cohort (neither Scenario I, nor Scenario II).

The model statistics for all covariates (aHR, 95% CI, and *p-*value) in multivariable Cox models were shown in Tables S3 (cutoff using quartiles) and S4 (cutoff using median). After performing the PSM method, the sample size of matched sub-cohort by early-timing versus delayed-timing was 3048 from the SEER-Medicare. The impact of delayed-timing as opposed to early-timing on GBM survival remained significant after repeating the Cox modeling in the PSM matched subsamples (Table S9, total patients after PSM).

### NCDB dataset

A total of 8161 GBM patients were included after exclusions, with a median time to AT at 33 days (range: 0–91 days). The median age at diagnosis was 71 years (IQR: 67–75, range: 65–90) and most of patients were Caucasians (90.0%), residents in metropolitan areas (81.7%), treated at academic centers (45.6%), and Medicare beneficiaries (82.1%). They were treated at academic facilities (45.6%) and were not referred (69.5%). In Table 2, the distributions of all the covariates except gender, insurance, and Charlson/Deyo Score varied significantly across times to start AT. Patients who were diagnosed from 2007 to 2014, blacks and Hispanics, treated at academic facilities, referred, and underwent resection were more likely to receive AT 27 days or later. In addition, patients treated at facilities in the South, Midwest, and Western areas of the USA, had a reduced likelihood of delayed AT compared to those treated at Northeast facilities (Table S8).

**Table 2:** Patient characteristics in NCDB analytic cohort by time to AT (N = 8161).

Characteristics, N (%)	Time to AT				
	≤ 15 days	16–26 days	27–37 days	≥ 38 days	P*
Patients	520	1868	2957	2816	
Socio-demographics					
Age at diagnosis, median (IQR)	72 (68, 77)	71 (67, 75)	71 (67, 75)	71 (67, 75)	0.002
Age at diagnosis, years					0.047
65–74	339 (65.2%)	1314 (70.3%)	2094 (70.8%)	2006 (71.2%)	
75–90	181 (34.8%)	554 (29.7%)	863 (29.2%)	810 (28.8%)	
Gender					0.470
Male	290 (55.8)	1096 (58.7)	1676 (56.7)	1602 (56.9)	
Female	230 (44.2)	772 (41.3)	1281 (43.3)	1214 (43.1)	
Year of diagnosis					< 0.001
1/2004–12/2006	132 (25.4)	343 (18.4)	371 (12.5)	319 (11.3)	
1/2007–12/2008	106 (20.4)	276 (14.8)	337 (11.4)	317 (11.3)	
1/2009–12/2010	96 (18.5)	324 (17.3)	519 (17.6)	482 (17.1)	
1/2011–12/2012	87 (16.7)	423 (22.6)	738 (25.0)	738 (26.2)	
1/2013–12/2014	99 (19.0)	502 (26.9)	992 (33.5)	960 (34.1)	
Race/ethnicity					< 0.001
White	487 (93.7)	1708 (91.4)	2681 (90.7)	2471 (87.7)	
Black	13 (2.5)	61 (3.3)	106 (3.6)	138 (4.9)	
Hispanic	NA	47 (2.5)	113 (3.8)	143 (5.1)	
Others	NA	52 (2.8)	57 (1.9)	64 (2.3)	
Residential location					0.015
Metropolitan	402 (77.3)	1519 (81.3)	2410 (81.5)	2339 (83.1)	
Urban/rural	118 (22.7)	349 (18.7)	547 (18.5)	477 (16.9)	
Education, %					< 0.001
≥ 29	79 (15.2)	194 (10.4)	324 (11.0)	406 (14.4)	
20–28.9	124 (23.8)	376 (20.1)	602 (20.4)	647 (23.0)	
14–19.9	132 (25.4)	476 (25.5)	761 (25.7)	693 (24.6)	
< 14	185 (35.6)	822 (44.0)	1270 (42.9)	1070 (38.0)	
Income, dollars					< 0.001
< 30,000	66 (12.7)	162 (8.7)	264 (8.9)	300 (10.7)	
30,000–35,999	101 (19.4)	311 (16.6)	500 (16.9)	487 (17.3)	
36,000–45,999	166 (31.9)	517 (27.7)	799 (27.0)	793 (28.2)	
≥ 46,000	187 (36.0)	878 (47.0)	1394 (47.1)	1236 (43.9)	
Insurance status					0.190
Not insured/medicaid/other government	NA	42 (2.2)	87 (2.9)	74 (2.6)	
Private insurance	NA	287 (15.4)	446 (15.1)	449 (15.9)	
Medicare	447 (86.0)	1539 (82.4)	2424 (82.0)	2293 (81.4)	
Facility characteristics					
Facility location					< 0.001
Northeast	80 (15.4)	370 (19.8)	636 (21.5)	750 (26.6)	
South	206 (39.6)	593 (31.7)	956 (32.3)	941 (33.4)	
Midwest	144 (27.7)	610 (32.7)	913 (30.9)	728 (25.9)	
West	90 (17.3)	295 (15.8)	452 (15.3)	397 (14.1)	
Facility type					0.002
Non-AC	259 (49.8)	821 (44.0)	1219 (41.2)	1164 (41.3)	
AC	200 (38.5)	818 (43.8)	1369 (46.3)	1337 (47.5)	
INCP	61 (11.7)	229 (12.3)	369 (12.5)	315 (11.2)	
Residence-hospital distance, miles					0.002
≤ 10	214 (41.2)	836 (44.8)	1250 (42.3)	1195 (42.4)	
(10–20]	84 (16.2)	392 (21.0)	669 (22.6)	613 (21.8)	
(20–50]	121 (23.3)	384 (20.6)	653 (22.1)	622 (22.1)	
> 50	101 (19.4)	256 (13.7)	385 (13.0)	386 (13.7)	
Care transition					< 0.001
None	438 (84.2)	1446 (77.4)	2107 (71.3)	1678 (59.6)	
Yes	82 (15.8)	422 (22.6)	850 (28.7)	1138 (40.4)	
Clinical treatments					
Surgery					< 0.001
Biopsy	113 (21.7)	328 (17.6)	437 (14.8)	438 (15.6)	
Resection	407 (78.3)	1540 (82.4)	2520 (85.2)	2378 (84.4)	
AT					< 0.001
Non-CRT	264 (50.8)	311 (16.6)	350 (11.8)	388 (13.8)	
CRT	256 (49.2)	1557 (83.4)	2607 (88.2)	2428 (86.2)	
Charlson/Deyo score					0.910
0	353 (67.9)	1297 (69.4)	2044 (69.1)	1923 (68.3)	
1	101 (19.4)	354 (19.0)	569 (19.2)	570 (20.2)	
≥ 2	66 (12.7)	217 (11.6)	344 (11.6)	323 (11.5)	

*AT* adjuvant therapy, *AC* academic center, *INCP* integrated network cancer programs, *RT* radiation therapy, *CRT* chemoradiation, *TMZ* temozolomide, *NA* not available, was presented once the number of patients in a specific cell was 11 or less based on privacy policy of NCDB.

*Pearson's χ^2^ test or Mann–Whitney *U* test were conducted to compare the proportions of baseline characteristics across time to AT.

As presented in Table 3, the median OS were 6.4, 9.8, 11.2, and 10.8 months corresponding to their respective timing groups for total patients (*P* < 0.001). For patients who underwent biopsy, the median OSs were prolonged from ≤ 15 days to ≥ 38 days (6.3, 7.2, 9.3, and 11.1 months, respectively, *P* = 0.003). Patients (resection group) who received AT between 27 and 37 days (11.4 months) following diagnosis achieved the longest OS compared to the remaining three intervals (≤ 15 days: 6.4, 16–26 days: 10.2, and ≥ 38 days: 10.8, months; *P* < 0.001). Figures 1D,E,F and 2D,E,F display Kaplan–Meier plots of survival curves for the NCDB cohort.

**Table 3:** Median OS, 1-year, and 2-year survival rates by time to AT and surgical procedure based on SEER-Medicare and NCDB.

Survival statistics	Time to AT				
	≤ 15 days	16–26 days	27–37 days	≥ 38 days	*P*
SEER-Medicare, N = 3159					
Total					< 0.001
Age at diagnosis, years (median, IQR)	73 (69–78)	73 (69–77)	72 (68–77)	73 (69–77)	
Death/total, N (%)	777/805 (96.5)	795/830 (95.8)	719/766 (93.9)	722/758 (95.3)	
Median OS (months, IQR)	5 (2–11)	6 (3–12)	6 (3–14)	7 (4–14)	
1-year survival rate, % (95% CI)	21.5 (18.7–24.4)	24.3 (21.5–27.3)	29.5 (26.3–32.8)	28.2 (25.1–31.5)	
2-year survival rate, % (95% CI)	8.0 (6.1–10.1)	8.6 (6.7–10.8)	9.4 (7.4–11.7)	9.9 (7.8–12.3)	
Biopsy					0.060
Age at diagnosis, years (median, IQR)	74 (70–79)	74 (70–79)	72.5 (68–78)	73 (69–79)	
Death/total, N (%)	348/354 (98.3)	333/339 (98.2)	267/276 (96.7)	256/263 (97.3)	
Median OS (months, IQR)	4 (2–9)	4 (2–9)	4 (2–8)	5 (3–10)	
1-year survival rate, % (95% CI)	17.2 (13.5–21.4)	15.3 (11.7–19.4)	17.0 (12.9–21.7)	20.2 (15.5–25.2)	
2-year survival rate, % (95% CI)	5.7 (3.5–8.6)	5.1 (3.1–8.0)	5.1 (2.9–8.3)	5.8 (3.3–9.3)	
Resection					< 0.001
Age at diagnosis, years (median, IQR)	73 (69–78)	72 (69–77)	72 (68–77)	72 (69–77)	
Death/TOTAL, N (%)	429/451 (95.1)	462/491 (94.1)	452/490 (92.2)	466/495 (94.1)	
Median OS (months, IQR)	6 (3–12)	7 (4–15)	9 (4–18)	8 (4–15)	
1-year survival rate, % (95% CI)	24.8 (20.9–28.9)	30.6 (26.5–34.7)	36.5 (32.3–40.8)	32.5 (28.4–36.7)	
2-year survival rate, % (95% CI)	9.8 (7.1–13.0)	11.2 (8.4–14.4)	11.9 (9.1–15.2)	12.1 (9.3–15.4)	
NCDB, N = 8161					
Total					< 0.001
Age at diagnosis, years (median, IQR)	72 (68–77)	71 (67–75)	71 (67–75)	71 (67–75)	
Death/total, N (%)	481/520 (92.5)	1736/1868 (92.9)	2661/2957 (90)	2541/2816 (90.2)	
Median OS (months, IQR)	6.4 (3.1–14.6)	9.8 (5.1–17.4)	11.2 (6.0–19.8)	10.8 (6.3–19.4)	
1-year survival rate, % (95%CI)	30.8 (26.8–34.8)	41.5 (39.2–43.7)	46.4 (44.6–48.2)	45.6 (43.7–47.5)	
2-year survival rate, % (95%CI)	10.3 (7.8–13.3)	14.7 (13–16.5)	18.1 (16.6–19.7)	17.6 (16.1–19.2)	
Biopsy					0.003
Age at diagnosis, years (median, IQR)	72 (68–77)	71 (68–76)	71 (67–76)	70 (67–74)	
Death/total, N (%)	102/113 (90.3)	300/328 (91.5)	396/437 (90.6)	396/438 (90.4)	
Median OS (months, IQR)	6.3 (3.2–14.3)	7.2 (3.9–16.0)	9.3 (4.6–16.5)	11.1 (6.0–19.2)	
1-year survival rate, % (95%CI)	29.4 (21.2–38.0)	36.2 (30.9–41.4)	39.4 (34.8–44.0)	47.7 (42.8–52.3)	
2-year survival rate, % (95%CI)	12.5 (6.7–20.3)	13.1 (9.3–17.6)	13.6 (10.3–17.5)	18.7 (14.7–23.0)	
Resection					< 0.001
Age at diagnosis, years (median, IQR)	72 (68–77)	71 (67–75)	71 (67–75)	71 (67–76)	
Death/total, N (%)	379/407 (93.1)	1436/1540 (93.3)	2265/2520 (89.9)	2145/2378 (90.2)	
Median OS (months, IQR)	6.4 (3.1–14.6)	10.2 (5.5–17.7)	11.4 (6.3–20.5)	10.8 (6.3–19.4)	
1-year survival rate, % (95% CI)	31.2 (26.7–35.7)	42.6 (40.1–45.1)	47.6 (45.6–49.6)	45.2 (43.2–47.2)	
2-year survival rate, % (95% CI)	9.8 (7.1–13.1)	15.1 (13.2–17.0)	18.9 (17.2–20.6)	17.5 (15.8–19.2)	

*AT* adjuvant therapy, *OS* overall survival, *IQR* interquartile range, *95% CI* 95% confidence interval.

The difference of Kaplan–Meier survival proportions across time to AT was tested by using two-sided log-rank test.

For the total cohort, the adjusted HRs indicated that the risk of death decreased by 17% in patients who received AT after a modest delay (27–37 days) from diagnosis (aHR 0.83, 95% CI 0.75–0.92, *P* < 0.001) and decreased by 16% in patients with the longest delay (≥ 38 days) (aHR 0.84, 95% CI 0.77–0.94, *P* = 0.002) compared to the shortest delay group (≤ 15 days). For the biopsy cohort, the risk of death for patients received AT ≥ 38 days was reduced by 23% in contrast to those in the group of ≤ 15 days (aHR 0.77, 95% CI 0.61–0.97, *P* = 0.024). For the resection group, patients with modest delay and the longest delay had 17% (aHR 0.83, 95% CI 0.74–0.93, *P* = 0.001) and 13% (aHR 0.87, 95% CI 0.77–0.98, *P* = 0.017) reduction in the risk of mortality, respectively (Table 4). The detailed model statistics for all covariates (aHR 95% CI and *P* value) are shown in Tables S5 and S6. Similar findings were observed in Scenario II (≥ 27 days vs. < 27 days): Biopsy, aHR 0.84, 95% CI 0.74–0.95, *P* = 0.006; Resection, aHR 0.89, 95% CI 0.84–0.94, *P* < 0.001; Total patients, aHR 0.88, 95% CI 0.83–0.93, *P* < 0.001 (Table 4) and in the PSM-matched sub-cohort (Table S9). After performing the PSM method, the sample size of matched sub-cohort by early-timing versus delayed-timing was 4776 from the NCDB.

**Table 4:** Multivariable Cox models of OS in relation to time to AT from SEER-Medicare and NCDB^a,b^.

Predictors	Biopsy (N = 1232)			Resection (N = 1927)			Total (N = 3159)		
	aHR	95%CI	*P*	aHR	95%CI	*P*	aHR	95%CI	*P*
SEER-Medicare, N = 3159^a^									
Time to AT, days									
≤ 15	1.00	–	–	1.00	–	–	1.00	–	–
16–26	1.03	0.88–1.20	0.706	0.90	0.78–1.02	0.105	0.97	0.87–1.07	0.496
27–37	1.04	0.89–1.23	0.608	0.74	0.64–0.84	< 0.001	0.86	0.78–0.96	0.005
≥ 38	0.86	0.73–1.01	0.068	0.81	0.71–0.92	0.002	0.86	0.78–0.95	0.004
Time to AT, cutoff as 27 days									
< 27	1.00	–	–	1.00	–	–	1.00	–	–
≥ 27	0.93	0.83–1.05	0.225	0.82	0.74–0.90	< 0.001	0.88	0.82–0.94	< 0.001

Predictors	Biopsy (N = 1316)			Resection (N = 6845)			Total (N = 8161)		
	aHR	95%CI	*P*	aHR	95%CI	*P*	aHR	95%CI	*P*
NCDB, N = 8161^b^									
Time to AT, days									
≤ 15	1.00	–	–	1.00	–	–	1.00	–	–
16–26	0.95	0.75–1.20	0.676	0.95	0.84–1.06	0.350	0.95	0.85–1.05	0.321
27–37	0.85	0.68–1.06	0.153	0.83	0.74–0.93	0.001	0.83	0.75–0.92	< 0.001
≥ 38	0.77	0.61–0.97	0.024	0.87	0.77–0.98	0.017	0.85	0.77–0.94	0.002
Time to AT, cutoff as 27 days									
< 27	1.00	–	–	1.00	–	–	1.00	–	–
≥ 27	0.84	0.74–0.95	0.006	0.89	0.84–0.94	< 0.001	0.88	0.83–0.93	< 0.001

*AT* adjuvant therapy, *HR* hazard ratio, *95% CI* 95% confidence interval.

^a^Adjusted time to AT, age at diagnosis, gender, period, race/ethnicity, marital status, residence, education, income, registry location, surgery, AT, and Charlson Comorbidity Score by using multivariable Cox proportional models in SEER-Medicare.

^b^Adjusted time to AT, age at diagnosis, gender, period, race/ethnicity, residence, education, income, insurance, facility location, distance, care transition, surgery, AT, and Charlson/Deyo score by using multivariable Cox proportional models in NCDB.

## Discussion

We explored the optimal timing of AT in elderly GBM patients using two large-scale national datasets. Our findings demonstrated that elderly GBM patients who underwent craniotomies and then received AT with a modest delay and a longer delay experienced a superior OS compared to those who were treated with AT immediately after surgery. After determining delayed-timing and early-timing using the cut-off of 27 days (median) (Scenario II), the survival benefit remained significant for patients received AT 27 days or later. More than 80% of patients received AT less than 2-month in the SEER-Medicare (80.6%) and the NCDB (83.3%). Significant predictors related to delayed timing (≥ 27 days) included the extent of resection (EoR), year of diagnosis, race, facility location, facility type, and care transition.

As shown in Table S1, prior studies revealed that various initiations within 4–6 weeks of AT might not be necessarily related to prolonged GBM survival based on RCT data^10,11^, public-accessible datasets^12,13,15,16^, data from health insurers^14^, and single-hospital/multi-center electronic health records (EHR)^17,18^. Those studies applied different thresholds to classify AT timing groups, including continuous form^18^, median^15,16,18^, tertiles^17^, quartiles^10,12,15,16,18^, incremental waiting days by 6 days from 15 to 42 days^13^, incremental waiting days by 2 weeks from 4 to 13 weeks^14^, and significant cut-off defined based on previous findings (42 days)^15^. However, the conclusions derived from these studies remain controversial.

Among all prior studies, only Lai et al. explored the association between RT timing and survival for elderly GBM patients in the SEER-Medicare dataset (1991–2002)^16^, which was before the era of the Stupp protocol^2^. They found no significant survival difference across the timing groups based on quartiles and median. Compared to the RTOG-based secondary analysis^10^ and the study using the Clinformatics Data Mart Database^14^, our study had a similar cut-off of timing groups and subsequent findings. Blumenthal et al. classified RT timing using quartiles and identified a 3.3-month survival benefit for patients with longer delay of RT initiation (> 4 weeks) over those received RT early (0–2 weeks) (median OS across Q1–Q4: 9.2, 10.8, 11.7, and 12.5 months, *P* < 0.001)^10^. The explanation of better median OSs results across four intervals in the RTOG-based clinical trial study than the OSs reported in this study is likely related to discrepancies between clinical trial participants and the general GBM population, as reported by many others^23^. Furthermore, a health insurer study in the U.S. demonstrated that CRT beginning within 4 weeks of craniotomy was associated with significantly inferior survival as compared to the middle delayed group (4–6 weeks) among HGG patients diagnosed between 2005 and 2014^14^. However, the study included not only GBM, but also grade III anaplastic astrocytoma and oligodendroglioma.

Based on the NCDB dataset, two studies investigated the effect of AT timing on GBM survival^12,13^. Osborn et al. identified that initiation of CCRT during 31–37 days was associated with improved GBM survival by 7% versus the reference group (0–24 days)^12^. However, they included GBM patients only diagnosed between 2010 and 2012, and excluded those underwent biopsies or received non-CRT and sequential CRT (SCRT). Pollom et al. categorized AT timing into six groups using an incremental of 6 days from 15 to 42 days and stratified the survival analysis by EoR^13^. Their results indicated that survival patterns varied across CRT timing groups by biopsy [< 15 days vs. > 42 days (ref.): HR, 1.67, *P* < 0.001] and resection [15–21 days vs. > 42 days: HR 0.82, *P* = 0.030]^13^. This study excluded GBM patients diagnosed from 2005 to 2009, which is similar to the exclusion criteria of Osborn et al. study. Considering the Stupp protocol was initiated in 2005, the removal of GBM patients diagnosed from 2005 to 2009 might lead to inaccurate estimates of time to AT and restrict the findings limited to a sub-population of treated with Stupp protocol.

Four pilot studies with limited sample sizes had generated contrary conclusions, such as moderate delay of CCRT (30–34 days over 0–30 days) improve GBM survival^11^, no survival difference across timing groups^17,18^, and longer delay (> 42 days vs. 0–42 days) decreased GBM survival^15^. In short, based on prior studies, the optimal timing of GBM AT still remain unclear due to lack of RCT data, limited sample sizes^11,15,17,18^, restricted database^10^, truncated period of GBM diagnosis^12,13^, lack of stratification by EoR^10,11,15,17,18^. Those publications have reported contrary conclusions (e.g., Sun et al.  > 42 days related to worse survival than 0–42 days^15^; Pollom et al.  > 42 days related to better survival than 15–21 days^13^).

Additionally, only three studies explored the predictors of delayed AT timing. Significant factors associated with delayed initiation of AT included blacks^12,13^, tumor size > 3 cm^12^, EoR^12,13,16^, treatment at academic facilities^12^, residence in metropolitan areas^13^, distance between residence-hospital > 50 miles^13^, and Medicaid/non-insured/other government insurance^13^. Only the highest tier of income (≥ $48,000) was reported as a negative factor^13^. In this study, we detected the higher likelihood of delayed AT timing is associated with surgical resection, later years of diagnosis, blacks and Hispanics, treatment at academic facilities, and receipt of referral in the NCDB database. From the SEER-Medicare dataset, we found only STR/GTR as the positive factor associated with delayed-timing, which agrees with the conclusion by Lai et al. study^16^.

Although the mechanisms underlying the survival advantage of moderate delayed time to AT still remain unknown, the potential explanation to the survival advantage of moderate delayed time to AT might be related to the transient brain tumor/tissue removal/injury and subsequent recovery from surgical procedures. Hypoxia occurs due to vasculature disruption from surgical resection in the remaining brain tissue around the surgical area, which may reduce sensitivity of residual GBM cells to RT and TMZ^24,25^. Regarding the mechanisms of RT on brain tumors, ionizing radiation produces organic free radicals that form only in the presence of oxygen, which then results in DNA damage. Therefore, the efficacy of RT may be diminished since tumor DNA in hypoxic environments are less succeptible^26^. Peker et al. suggested that the rats that received RT within 1–2 weeks following brain surgery had significantly higher levels of tissue damage compared to those started RT ≥ 3 weeks after operation^27^. We speculate that early initiation of AT may diminish the killing capacity to GBM tumor cells by the RT and TMZ in a possible hypoxic environment, resulting in shorter patients’ survival. In addition, for patients underwent biopsy only, these patients usually were in poor health status or, tumor located in eloquent areas. The significant survival benefit of delayed AT on biopsy only group in the NCDB, but not in the SEER-Medicare dataset could be due to the differences in these two datasets on target population (SEER-Medicare: population-based data; NCDB: hospital-based data), data sources (SEER-Medicare: 18 cancer registries; NCDB: over 1500 CoC-accredited facilities), and proportion of general population (SEER-Medicare: covering elderly cancer patients from around 34% of the U.S. population; NCDB: around 70% of newly diagnosed cancer cases). Also, patients underwent biopsy only may be more likely to receive standard RT or shortened hypofractionated RT, which may make the mechanisms underlying the survival advantage of delayed AT more complex.

The present study is the first to comprehensively explore the optimal timing of AT for elderly GBM based on the SEER-Medicare dataset with a validation using the NCDB dataset. Also, we validated two classification methods of time to AT (quartiles or median) and obtained consistent results. Further, our analysis included the first full coverage of the Stupp protocol since 2005 and even extended one year prior to 2005 (SEER-Medicare: 2004–2013; NCDB: 2004–2014), which could cover the potential off-label usage of TMZ as well. Besides multivariable Cox proportional hazards model and stratification by biopsy and resection, we performed 1:1 PSM method as sensitivity analysis to minimize the impact of potential confounders or selection bias.

There are several limitations in the present study. First, our study is retrospective analyses of two large datasets including large proportion of elderly GBM patients, which might introduce selection bias or confounders. To minimize the impact of confounders/bias, we performed multivariable models, stratifications, and PSM method. However, PSM could not address the imbalance of unknown or unmeasured variables across timing groups. Second, the survival benefit detected in patients with delayed AT might be related to survival bias, a time-dependent variable for AT initiation which could be helpful to minimize the potential impact of survival bias in future studies. Lastly, there is no data provided regarding molecular profiles [e.g., isocitrate dehydrogenase1/2 (*IDH*1/2)^28–30^, or *O*^6^-methylguanine DNA methyltransferase (*MGMT*)^31–33^], the percent of resection or residual tumor volume^34–40^, Karnofsky Performance Scale (KPS)^36,41,42^, which may influence our findings on OS.

## Conclusions

For the best timing in elderly GBM patients to start AT, superior survivals were observed among those who had craniotomy and initiated AT with a modest (27–37 days) or longer delay (≥ 38 days) following diagnosis based on analysis from both SEER-Medicare and NCDB datasets (Scenario I). Such survival advantage was confirmed in delayed-timing group in both datasets (Scenario II). The increased likelihood of receiving delayed AT (≥ 27 days) was significantly associated with tumor resection (STR/GTR), years of diagnosis after 2006, African American and Hispanics races, treatments at academic facilities, and being referred. There is no difference in timing of AT on survival among elderly GBM patients who had biopsy in the SEER-Medicare dataset. We conclude that initiating AT with a modest delay (27–37 days) or a longer delay (≥ 38 days) after craniotomy may be the preferred timing in the elderly GBM population.

## Supplementary Information

Supplementary Figures.

Supplementary Tables.

## Data Availability

The datasets used and/or analyzed during the current study are available from the National Cancer Institute and American College of Surgeons (https://www.facs.org/quality-programs/cancer-programs/).
