# Supplementary material for: Impact of timing to initiate adjuvant therapy on survival of elderly glioblastoma patients using the SEER-Medicare and national cancer databases
Source: Sci Rep. 2023 Feb 25;13:3266. doi: 10.1038/s41598-023-30017-z (PMC9968296; doi:10.1038/s41598-023-30017-z)
Supplement: Supplementary file 1 — Supplementary Figures. [file 41598_2023_30017_MOESM1_ESM.docx]

*Impact of Timing to Initiate Adjuvant Therapy on Survival of Elderly Glioblastoma Patients:*

*Findings from Large Cohorts of SEER-Medicare and National Cancer Database*

**Figures and Captions**

**Figure S1.** Flow diagram of eligible GBM patients derived from SEER-Medicare and NCDB.

**Figure. S1. Flow diagram of eligible GBM patients data derived from NCDB (A) and SEER-Medicare (B).**

GBM, glioblastoma; NCDB, National Cancer Data Base; SEER-Medicare, Surveillance, Epidemiology and End Results (SEER)-Medicare linked dataset; Participant Use File; RT, radiotherapy.

*P*=0.003

*P*<0.001

*P*<0.001

**Figure 1**. Overall survival of GBM patients across time to adjuvant therapy (four-category) by applying the Kaplan-Meier method*.

*: “-” was presented once the number of patients was 11 or less based on privacy policy of both SEER-Medicare and NCDB.

A: Overall survival of GBM across time to adjuvant therapy in patients undergoing biopsy from SEER-Medicare.

B: Overall survival of GBM across time to adjuvant therapy in patients undergoing resection from SEER-Medicare.

C: Overall survival of GBM across time to adjuvant therapy among total patients from SEER-Medicare.

D: Overall survival of GBM across time to adjuvant therapy in patients undergoing biopsy from NCDB.

E: Overall survival of GBM across time to adjuvant therapy in patients undergoing resection from NCDB.

F: Overall survival of GBM across time to adjuvant therapy among total patients from NCDB.

*P*<0.001

*P*<0.001

*P*=0.003

*P*<0.001

*P*<0.001

*P*=0.003

*P*<0.001

*P*<0.001

*P*=0.003
