# Supplementary material for: Impact of timing to initiate adjuvant therapy on survival of elderly glioblastoma patients using the SEER-Medicare and national cancer databases
Source: Sci Rep. 2023 Feb 25;13:3266. doi: 10.1038/s41598-023-30017-z (PMC9968296; doi:10.1038/s41598-023-30017-z)
Supplement: Supplementary file 2 — Supplementary Tables. [file 41598_2023_30017_MOESM2_ESM.docx]

*Impact of Timing to Initiate Adjuvant Therapy on Survival of Elderly Glioblastoma Patients:*

*Findings from Large Cohorts of SEER-Medicare and National Cancer Database*

**Tables and Captions**

*Supplementary Table S1-Table S9*

**Table S1**. Comparison of the RCT secondary analysis, population-based setting, and single-institution studies on time to adjuvant therapy and GBM outcomes. (N=9)

**Table S2**. Definition and coding for GBM, basic characteristics, treatment, and outcomes.

**Table S3**. Multivariable Cox models of OS in relation to time to adjuvant therapy from SEER-Medicare. (Four-category)

**Table S4**. Multivariable Cox models of OS in relation to time to adjuvant therapy from SEER-Medicare. (Two-category)

**Table S5**. Multivariable Cox models of OS in relation to time to adjuvant therapy from NCDB*. (Four-category)

**Table S6**. Multivariable Cox models of OS in relation to time to adjuvant therapy from NCDB*. (Two-category)

**Table S7.** Likelihood of delayed timing vs. early timing and the related factors in SEER-Medicare. (N=3159)

**Table S8.** Likelihood of delayed timing versus early timing and the related factors in NCDB. (N=8161)

**Table S9**. Multivariable Cox models of OS for the PSM matched cohort from SEER-Medicare and NCDB.

**Table S2**. Definition and coding for GBM, basic characteristics, treatment, and outcomes.

| **Table S3**. Multivariable Cox models of OS in relation to time to adjuvant therapy from SEER-Medicare*. (Four-category) | | | | | | | | | | | |  |
| --- | --- | --- | --- | --- | --- | --- | --- | --- | --- | --- | --- | --- |
|  | Biopsy (N=1232) | | |  | Resection (N=1927) | | |  | Total (N=3159) | | |  |
| Predictors | aHR | 95%CI | *P* |  | aHR | 95%CI | *P* |  | aHR | 95%CI | *P* |  |
| **Time to adjuvant therapy, days** |  |  |  |  |  |  |  |  |  |  |  |  |
| ≤15 | 1.00 | - | - |  | 1.00 | - | - |  | 1.00 | - | - |  |
| 16-26 | 1.03 | 0.88 - 1.20 | 0.706 |  | 0.90 | 0.78 - 1.02 | 0.105 |  | 0.97 | 0.87 - 1.07 | 0.496 |  |
| 27-37 | 1.04 | 0.89 - 1.23 | 0.608 |  | 0.74 | 0.64 - 0.84 | <0.001 |  | 0.86 | 0.78 - 0.96 | 0.005 |  |
| ≥38 | 0.86 | 0.73 - 1.01 | 0.068 |  | 0.81 | 0.71 - 0.92 | 0.002 |  | 0.86 | 0.78 - 0.95 | 0.004 |  |
| **Socio-demographics** |  |  |  |  |  |  |  |  |  |  |  |  |
| Age at diagnosis, years |  |  |  |  |  |  |  |  |  |  |  |  |
| 65-74 | 1.00 | - | - |  | 1.00 | - | - |  | 1.00 | - | - |  |
| 75-90 | 1.20 | 1.06 - 1.36 | 0.003 |  | 1.27 | 1.15 - 1.41 | <0.001 |  | 1.25 | 1.15 - 1.35 | <0.001 |  |
| Gender |  |  |  |  |  |  |  |  |  |  |  |  |
| Male | 1.00 | - | - |  | 1.00 | - | - |  | 1.00 | - | - |  |
| Female | 1.01 | 0.89 - 1.14 | 0.889 |  | 1.00 | 0.91 - 1.10 | 0.985 |  | 1.00 | 0.92 - 1.08 | 0.936 |  |
| Year of diagnosis |  |  |  |  |  |  |  |  |  |  |  |  |
| 1/2004-12/2005 | 1.00 | - | - |  | 1.00 | - | - |  | 1.00 | - | - |  |
| 1/2006-12/2007 | 0.99 | 0.83 - 1.17 | 0.870 |  | 1.05 | 0.91 - 1.21 | 0.487 |  | 1.01 | 0.91 - 1.12 | 0.869 |  |
| 1/2008-12/2009 | 0.92 | 0.78 - 1.10 | 0.375 |  | 0.95 | 0.82 - 1.10 | 0.484 |  | 0.92 | 0.82 - 1.03 | 0.133 |  |
| 1/2010-12/2011 | 1.04 | 0.87 - 1.24 | 0.661 |  | 1.02 | 0.90 - 1.17 | 0.725 |  | 1.03 | 0.92 - 1.14 | 0.624 |  |
| 1/2012-12/2014 | 1.19 | 0.93 - 1.51 | 0.165 |  | 0.91 | 0.77 - 1.08 | 0.300 |  | 0.98 | 0.85 - 1.12 | 0.722 |  |
| Race/Ethnicity |  |  |  |  |  |  |  |  |  |  |  |  |
| White | 1.00 | - | - |  | 1.00 | - | - |  | 1.00 | - | - |  |
| Black | 0.79 | 0.58 - 1.09 | 0.151 |  | 0.57 | 0.43 - 0.75 | <0.001 |  | 0.65 | 0.53 - 0.80 | <0.001 |  |
| Hispanic | 0.81 | 0.51 - 1.28 | 0.365 |  | 0.69 | 0.45 - 1.06 | 0.089 |  | 0.77 | 0.56 - 1.04 | 0.091 |  |
| Others | 0.67 | 0.49 - 0.90 | 0.009 |  | 0.77 | 0.58 - 1.02 | 0.067 |  | 0.73 | 0.59 - 0.90 | 0.003 |  |
| Marital status |  |  |  |  |  |  |  |  |  |  |  |  |
| Single/DWS | 1.00 | - | - |  | 1.00 | - | - |  | 1.00 | - | - |  |
| Married | 0.93 | 0.82 - 1.06 | 0.289 |  | 0.90 | 0.81 - 1.00 | 0.044 |  | 0.92 | 0.85 - 1.00 | 0.061 |  |
| Residential location |  |  |  |  |  |  |  |  |  |  |  |  |
| Metropolitan | 1.00 | - | - |  | 1.00 | - | - |  | 1.00 | - | - |  |
| Urban/Rural | 0.93 | 0.79 - 1.11 | 0.432 |  | 1.08 | 0.94 - 1.24 | 0.269 |  | 1.02 | 0.92 - 1.13 | 0.723 |  |
| Education, % |  |  |  |  |  |  |  |  |  |  |  |  |
| ≥29 | 1.00 | - | - |  | 1.00 | - | - |  | 1.00 | - | - |  |
| 20-28.9 | 1.03 | 0.83 - 1.28 | 0.768 |  | 1.03 | 0.85 - 1.24 | 0.785 |  | 1.03 | 0.89 - 1.18 | 0.716 |  |
| 14-19.9 | 1.11 | 0.88 - 1.41 | 0.378 |  | 1.04 | 0.86 - 1.28 | 0.670 |  | 1.08 | 0.92 - 1.25 | 0.341 |  |
| <14 | 0.97 | 0.77 - 1.24 | 0.832 |  | 0.96 | 0.79 - 1.17 | 0.697 |  | 0.98 | 0.84 - 1.14 | 0.768 |  |
| Income, dollars |  |  |  |  |  |  |  |  |  |  |  |  |
| <30,000 | 1.00 | - | - |  | 1.00 | - | - |  | 1.00 | - | - |  |
| 30,000-35,999 | 1.00 | 0.79 - 1.28 | 0.984 |  | 0.93 | 0.76 - 1.15 | 0.512 |  | 0.96 | 0.83 - 1.13 | 0.650 |  |
| 36,000-45,999 | 0.92 | 0.72 - 1.17 | 0.500 |  | 0.91 | 0.75 - 1.09 | 0.300 |  | 0.91 | 0.79 - 1.06 | 0.221 |  |
| ≥46,000 | 1.01 | 0.78 - 1.30 | 0.951 |  | 0.89 | 0.72 - 1.09 | 0.244 |  | 0.93 | 0.79 - 1.08 | 0.343 |  |
| **Facility characteristics** |  |  |  |  |  |  |  |  |  |  |  |  |
| Registry location |  |  |  |  |  |  |  |  |  |  |  |  |
| Northeast | 1.00 | - | - |  | 1.00 | - | - |  | 1.00 | - | - |  |
| North Central | 1.22 | 0.98 - 1.51 | 0.072 |  | 1.08 | 0.91 - 1.28 | 0.370 |  | 1.12 | 0.99 - 1.28 | 0.081 |  |
| South | 1.14 | 0.94 - 1.38 | 0.180 |  | 1.19 | 1.02 - 1.40 | 0.031 |  | 1.13 | 1.00 - 1.28 | 0.044 |  |
| West | 1.11 | 0.94 - 1.31 | 0.212 |  | 0.99 | 0.87 - 1.12 | 0.877 |  | 1.03 | 0.93 - 1.14 | 0.550 |  |
| Teaching status |  |  |  |  |  |  |  |  |  |  |  |  |
| No | 1.00 | - | - |  | 1.00 | - | - |  | 1.00 | - | - |  |
| Yes | 0.98 | 0.86 - 1.10 | 0.689 |  | 0.95 | 0.86 - 1.05 | 0.322 |  | 0.96 | 0.89 - 1.03 | 0.266 |  |
| **Clinical treatments** |  |  |  |  |  |  |  |  |  |  |  |  |
| Surgery |  |  |  |  |  |  |  |  |  |  |  |  |
| Biopsy | - | - | - |  | 1.00 | - | - |  | 1.00 | - | - |  |
| STR | - | - | - |  | 0.72 | 0.65 - 0.79 | <0.001 |  | 0.85 | 0.77 - 0.93 | <0.001 |  |
| GTR | - | - | - |  | - | - | - |  | 0.62 | 0.57 - 0.68 | <0.001 |  |
| Adjuvant therapy |  |  |  |  |  |  |  |  |  |  |  |  |
| Non-CRT | 1.00 | - | - |  | 1.00 | - | - |  | 1.00 | - | - |  |
| CRT | 0.57 | 0.50 - 0.65 | <0.001 |  | 0.50 | 0.45 - 0.56 | <0.001 |  | 0.54 | 0.49 - 0.58 | <0.001 |  |
| Charlson Comorbidity Score |  |  |  |  |  |  |  |  |  |  |  |  |
| 0 | 1.00 | - | - |  | 1.00 | - | - |  | 1.00 | - | - |  |
| 1 | 1.23 | 1.07 - 1.41 | 0.004 |  | 1.28 | 1.14 - 1.43 | <0.001 |  | 1.23 | 1.13 - 1.34 | <0.001 |  |
| ≥ 2 | 1.38 | 1.19 - 1.60 | <0.001 |  | 1.46 | 1.29 - 1.66 | <0.001 |  | 1.43 | 1.30 - 1.57 | <0.001 |  |
| Abbreviation: HR, hazard ratio; 95%CI, 95% confidence interval; STR, subtotal resection; GTR, gross total resection; CRT, chemoradiation. | | | | | | | | | | | | |
| *: Adjusted time to adjuvant therapy (four-category variable), age at diagnosis, gender, period, race/ethnicity, marital status, residence, education, income, registry location, surgery, adjuvant therapy, and Charlson Comorbidity Score by using multivariable Cox proportional models in SEER-Medicare. | | | | | | | | | | | | |

| **Table S4**. Multivariable Cox models of OS in relation to time to adjuvant therapy from SEER-Medicare*. (Two-category) | | | | | | | | | | | |
| --- | --- | --- | --- | --- | --- | --- | --- | --- | --- | --- | --- |
|  | Biopsy (N=1232) | | |  | Resection (N=1927) | | |  | Total (N=3159) | | |
| Predictors | aHR | 95%CI | *P* |  | aHR | 95%CI | *P* |  | aHR | 95%CI | *P* |
| **Time to adjuvant therapy, days** |  |  |  |  |  |  |  |  |  |  |  |
| <26 | 1.00 | - | - |  | 1.00 | - | - |  | 1.00 | - | - |
| ≥27 | 0.93 | 0.83 - 1.05 | 0.225 |  | 0.82 | 0.74 - 0.90 | <0.001 |  | 0.88 | 0.82 - 0.94 | <0.001 |
| **Socio-demographics** |  |  |  |  |  |  |  |  |  |  |  |
| Age at diagnosis, years |  |  |  |  |  |  |  |  |  |  |  |
| 65-74 | 1.00 | - | - |  | 1.00 | - | - |  | 1.00 | - | - |
| 75-90 | 1.20 | 1.06 - 1.36 | 0.004 |  | 1.27 | 1.15 - 1.41 | <0.001 |  | 1.25 | 1.15 - 1.35 | <0.001 |
| Gender |  |  |  |  |  |  |  |  |  |  |  |
| Male | 1.00 | - | - |  | 1.00 | - | - |  | 1.00 | - | - |
| Female | 1.01 | 0.89 - 1.14 | 0.864 |  | 1.00 | 0.91 - 1.10 | 0.982 |  | 1.00 | 0.92 - 1.08 | 0.936 |
| Year of diagnosis |  |  |  |  |  |  |  |  |  |  |  |
| 1/2004-12/2005 | 1.00 | - | - |  | 1.00 | - | - |  | 1.00 | - | - |
| 1/2006-12/2007 | 0.97 | 0.82 - 1.16 | 0.755 |  | 1.06 | 0.92 - 1.22 | 0.395 |  | 1.01 | 0.91 - 1.12 | 0.860 |
| 1/2008-12/2009 | 0.91 | 0.77 - 1.08 | 0.285 |  | 0.95 | 0.82 - 1.10 | 0.478 |  | 0.92 | 0.82 - 1.03 | 0.132 |
| 1/2010-12/2011 | 1.04 | 0.87 - 1.24 | 0.676 |  | 1.03 | 0.90 - 1.17 | 0.678 |  | 1.03 | 0.93 - 1.14 | 0.610 |
| 1/2012-12/2014 | 1.16 | 0.91 - 1.47 | 0.228 |  | 0.91 | 0.77 - 1.09 | 0.302 |  | 0.98 | 0.85 - 1.12 | 0.730 |
| Race/Ethnicity |  |  |  |  |  |  |  |  |  |  |  |
| White | 1.00 | - | - |  | 1.00 | - | - |  | 1.00 | - | - |
| Black | 0.77 | 0.56 - 1.06 | 0.104 |  | 0.56 | 0.43 - 0.74 | <0.001 |  | 0.65 | 0.53 - 0.80 | <0.001 |
| Hispanic | 0.79 | 0.50 - 1.26 | 0.328 |  | 0.69 | 0.45 - 1.06 | 0.090 |  | 0.76 | 0.56 - 1.04 | 0.089 |
| Others | 0.67 | 0.50 - 0.91 | 0.011 |  | 0.78 | 0.59 - 1.03 | 0.075 |  | 0.73 | 0.59 - 0.90 | 0.003 |
| Marital status |  |  |  |  |  |  |  |  |  |  |  |
| Single/DWS | 1.00 | - | - |  | 1.00 | - | - |  | 1.00 | - | - |
| Married | 0.94 | 0.82 - 1.07 | 0.335 |  | 0.90 | 0.81 - 1.00 | 0.056 |  | 0.92 | 0.85 - 1.00 | 0.062 |
| Residential location |  |  |  |  |  |  |  |  |  |  |  |
| Metropolitan | 1.00 | - | - |  | 1.00 | - | - |  | 1.00 | - | - |
| Urban/Rural | 0.94 | 0.79 - 1.12 | 0.481 |  | 1.08 | 0.94 - 1.24 | 0.288 |  | 1.02 | 0.92 - 1.13 | 0.718 |
| Education, % |  |  |  |  |  |  |  |  |  |  |  |
| ≥29 | 1.00 | - | - |  | 1.00 | - | - |  | 1.00 | - | - |
| 20-28.9 | 1.03 | 0.83 - 1.29 | 0.762 |  | 1.03 | 0.85 - 1.24 | 0.754 |  | 1.03 | 0.89 - 1.18 | 0.698 |
| 14-19.9 | 1.12 | 0.88 - 1.42 | 0.341 |  | 1.05 | 0.86 - 1.28 | 0.661 |  | 1.08 | 0.93 - 1.25 | 0.338 |
| <14 | 0.98 | 0.77 - 1.24 | 0.856 |  | 0.97 | 0.79 - 1.18 | 0.739 |  | 0.98 | 0.84 - 1.14 | 0.794 |
| Income, dollars |  |  |  |  |  |  |  |  |  |  |  |
| <30,000 | 1.00 | - | - |  | 1.00 | - | - |  | 1.00 | - | - |
| 30,000-35,999 | 0.99 | 0.78 - 1.26 | 0.930 |  | 0.93 | 0.75 - 1.14 | 0.478 |  | 0.96 | 0.83 - 1.13 | 0.644 |
| 36,000-45,999 | 0.90 | 0.71 - 1.15 | 0.405 |  | 0.90 | 0.75 - 1.09 | 0.270 |  | 0.91 | 0.79 - 1.06 | 0.215 |
| ≥46,000 | 0.99 | 0.77 - 1.28 | 0.952 |  | 0.87 | 0.71 - 1.07 | 0.192 |  | 0.93 | 0.79 - 1.08 | 0.331 |
| **Facility characteristics** |  |  |  |  |  |  |  |  |  |  |  |
| Registry location |  |  |  |  |  |  |  |  |  |  |  |
| Northeast | 1.00 | - | - |  | 1.00 | - | - |  | 1.00 | - | - |
| North Central | 1.22 | 0.99 - 1.51 | 0.066 |  | 1.08 | 0.91 - 1.28 | 0.366 |  | 1.12 | 0.99 - 1.28 | 0.080 |
| South | 1.13 | 0.93 - 1.37 | 0.211 |  | 1.19 | 1.01 - 1.40 | 0.034 |  | 1.13 | 1.00 - 1.28 | 0.043 |
| West | 1.11 | 0.94 - 1.31 | 0.207 |  | 0.99 | 0.88 - 1.13 | 0.930 |  | 1.03 | 0.94 - 1.14 | 0.530 |
| Teaching status |  |  |  |  |  |  |  |  |  |  |  |
| No | 1.00 | - | - |  | 1.00 | - | - |  | 1.00 | - | - |
| Yes | 0.97 | 0.86 - 1.09 | 0.586 |  | 0.95 | 0.86 - 1.05 | 0.351 |  | 0.96 | 0.89 - 1.03 | 0.265 |
| **Clinical treatments** |  |  |  |  |  |  |  |  |  |  |  |
| Surgery |  |  |  |  |  |  |  |  |  |  |  |
| Biopsy | - | - | - |  | - | - | - |  | 1.00 | - | - |
| STR | - | - | - |  | 1.00 | - | - |  | 0.85 | 0.77 - 0.93 | <0.001 |
| GTR | - | - | - |  | 0.72 | 0.65 - 0.79 | <0.001 |  | 0.62 | 0.57 - 0.68 | <0.001 |
| Adjuvant therapy |  |  |  |  |  |  |  |  |  |  |  |
| Non-CRT | 1.00 | - | - |  | 1.00 | - | - |  | 1.00 | - | - |
| CRT | 0.58 | 0.51 - 0.66 | <0.001 |  | 0.50 | 0.45 - 0.56 | <0.001 |  | 0.54 | 0.49 - 0.58 | <0.001 |
| Charlson Comorbidity Score |  |  |  |  |  |  |  |  |  |  |  |
| 0 | 1.00 | - | - |  | 1.00 | - | - |  | 1.00 | - | - |
| 1 | 1.22 | 1.06 - 1.40 | 0.005 |  | 1.28 | 1.14 - 1.43 | <0.001 |  | 1.23 | 1.13 - 1.35 | <0.001 |
| ≥ 2 | 1.38 | 1.19 - 1.59 | <0.001 |  | 1.46 | 1.29 - 1.66 | <0.001 |  | 1.43 | 1.30 - 1.57 | <0.001 |
| Abbreviation: HR, hazard ratio; 95%CI, 95% confidence interval; STR, subtotal resection; GTR, gross total resection; CRT, chemoradiation. | | | | | | | | | | | |
| a: Adjusted time to adjuvant therapy (two-category), age at diagnosis, gender, period, race/ethnicity, marital status, residence, education, income, registry location, surgery, adjuvant therapy, and Charlson Comorbidity Score by using multivariable Cox proportional models in SEER-Medicare. | | | | | | | | | | | |

| **Table S5**. Multivariable Cox models of OS in relation to time to adjuvant therapy from NCDB*. (Four-category) | | | | | | | | | | | |
| --- | --- | --- | --- | --- | --- | --- | --- | --- | --- | --- | --- |
|  | Biopsy (N=1316) | | |  | Resection (N=6845) | | |  | Total (N=8161) | | |
| Predictors | aHR | 95%CI | *P* |  | aHR | 95%CI | *P* |  | aHR | 95%CI | *P* |
| **Time to adjuvant therapy, days** |  |  |  |  |  |  |  |  |  |  |  |
| ≤15 | 1.00 | - | - |  | 1.00 | - | - |  | 1.00 | - | - |
| 16-26 | 0.95 | 0.75 - 1.20 | 0.676 |  | 0.95 | 0.84 - 1.06 | 0.350 |  | 0.95 | 0.85 - 1.05 | 0.321 |
| 27-37 | 0.85 | 0.68 - 1.06 | 0.153 |  | 0.83 | 0.74 - 0.93 | 0.001 |  | 0.83 | 0.75 - 0.92 | <0.001 |
| ≥38 | 0.77 | 0.61 - 0.97 | 0.024 |  | 0.87 | 0.77 - 0.98 | 0.017 |  | 0.85 | 0.77 - 0.94 | 0.002 |
| **Socio-demographics** |  |  |  |  |  |  |  |  |  |  |  |
| Age at diagnosis, years |  |  |  |  |  |  |  |  |  |  |  |
| 65-74 | 1.00 | - | - |  | 1.00 | - | - |  | 1.00 | - | - |
| 75-90 | 1.50 | 1.31 - 1.70 | <0.001 |  | 1.39 | 1.31 - 1.47 | <0.001 |  | 1.41 | 1.34 - 1.49 | <0.001 |
| Gender |  |  |  |  |  |  |  |  |  |  |  |
| Male | 1.00 | - | - |  | 1.00 | - | - |  | 1.00 | - | - |
| Female | 1.00 | 0.88 - 1.12 | 0.963 |  | 0.96 | 0.91 - 1.01 | 0.105 |  | 0.97 | 0.92 - 1.01 | 0.171 |
| Year of diagnosis |  |  |  |  |  |  |  |  |  |  |  |
| 1/2004-12/2006 | 1.00 | - | - |  | 1.00 | - | - |  | 1.00 | - | - |
| 1/2007-12/2008 | 1.16 | 0.81 - 1.64 | 0.415 |  | 0.96 | 0.88 - 1.05 | 0.375 |  | 0.97 | 0.89 - 1.06 | 0.514 |
| 1/2009-12/2010 | 1.08 | 0.82 - 1.42 | 0.595 |  | 0.98 | 0.90 - 1.06 | 0.574 |  | 0.97 | 0.90 - 1.05 | 0.501 |
| 1/2011-12/2012 | 1.05 | 0.81 - 1.37 | 0.691 |  | 0.90 | 0.83 - 0.98 | 0.014 |  | 0.91 | 0.84 - 0.98 | 0.015 |
| 1/2013-12/2014 | 1.19 | 0.91 - 1.54 | 0.197 |  | 0.88 | 0.81 - 0.95 | 0.001 |  | 0.91 | 0.85 - 0.98 | 0.015 |
| Race/Ethnicity |  |  |  |  |  |  |  |  |  |  |  |
| White | 1.00 | - | - |  | 1.00 | - | - |  | 1.00 | - | - |
| Black | 0.96 | 0.71 - 1.30 | 0.798 |  | 0.89 | 0.78 - 1.02 | 0.101 |  | 0.91 | 0.81 - 1.03 | 0.152 |
| Hispanic | 0.99 | 0.72 - 1.36 | 0.943 |  | 0.76 | 0.66 - 0.88 | <0.001 |  | 0.80 | 0.71 - 0.92 | 0.001 |
| Others | 0.80 | 0.54 - 1.17 | 0.247 |  | 0.76 | 0.63 - 0.91 | 0.003 |  | 0.75 | 0.64 - 0.88 | 0.001 |
| Residential location |  |  |  |  |  |  |  |  |  |  |  |
| Metropolitan | 1.00 | - | - |  | 1.00 | - | - |  | 1.00 | - | - |
| Urban/Rural | 1.06 | 0.88 - 1.28 | 0.506 |  | 1.01 | 0.93 - 1.10 | 0.778 |  | 1.02 | 0.95 - 1.10 | 0.607 |
| Education, % |  |  |  |  |  |  |  |  |  |  |  |
| ≥29 | 1.00 | - | - |  | 1.00 | - | - |  | 1.00 | - | - |
| 20-28.9 | 1.00 | 0.81 - 1.25 | 0.966 |  | 1.13 | 1.03 - 1.25 | 0.013 |  | 1.11 | 1.02 - 1.21 | 0.021 |
| 14-19.9 | 1.02 | 0.81 - 1.29 | 0.848 |  | 1.18 | 1.06 - 1.31 | 0.002 |  | 1.15 | 1.05 - 1.27 | 0.004 |
| <14 | 0.88 | 0.68 - 1.13 | 0.299 |  | 1.19 | 1.07 - 1.33 | 0.002 |  | 1.14 | 1.03 - 1.26 | 0.013 |
| Income, dollars |  |  |  |  |  |  |  |  |  |  |  |
| <30,000 | 1.00 | - | - |  | 1.00 | - | - |  | 1.00 | - | - |
| 30,000-35,999 | 1.11 | 0.87 - 1.40 | 0.403 |  | 0.97 | 0.87 - 1.08 | 0.611 |  | 1.00 | 0.91 - 1.10 | 0.959 |
| 36,000-45,999 | 1.18 | 0.93 - 1.49 | 0.173 |  | 0.91 | 0.82 - 1.02 | 0.106 |  | 0.96 | 0.87 - 1.06 | 0.398 |
| ≥46,000 | 1.08 | 0.83 - 1.41 | 0.577 |  | 0.83 | 0.73 - 0.93 | 0.002 |  | 0.87 | 0.78 - 0.97 | 0.009 |
| Insurance status |  |  |  |  |  |  |  |  |  |  |  |
| Not insured/Medicaid/Other government | 1.00 | - | - |  | 1.00 | - | - |  | 1.00 | - | - |
| Private insurance | 1.58 | 1.05 - 2.38 | 0.029 |  | 0.99 | 0.83 - 1.19 | 0.928 |  | 1.08 | 0.91 - 1.27 | 0.367 |
| Medicare | 1.61 | 1.09 - 2.37 | 0.017 |  | 1.03 | 0.87 - 1.22 | 0.750 |  | 1.12 | 0.95 - 1.30 | 0.173 |
| **Facility characteristics** |  |  |  |  |  |  |  |  |  |  |  |
| Facility location |  |  |  |  |  |  |  |  |  |  |  |
| Northeast | 1.00 | - | - |  | 1.00 | - | - |  | 1.00 | - | - |
| South | 1.22 | 1.03 - 1.44 | 0.019 |  | 1.12 | 1.04 - 1.21 | 0.002 |  | 1.13 | 1.06 - 1.21 | <0.001 |
| Midwest | 1.06 | 0.90 - 1.25 | 0.486 |  | 1.12 | 1.04 - 1.20 | 0.003 |  | 1.11 | 1.03 - 1.18 | 0.003 |
| West | 1.17 | 0.96 - 1.42 | 0.120 |  | 1.05 | 0.96 - 1.14 | 0.304 |  | 1.07 | 0.99 - 1.16 | 0.095 |
| Facility type |  |  |  |  |  |  |  |  |  |  |  |
| Non-AC | 1.00 | - | - |  | 1.00 | - | - |  | 1.00 | - | - |
| AC | 0.81 | 0.71 - 0.92 | 0.001 |  | 0.89 | 0.84 - 0.94 | <0.001 |  | 0.87 | 0.83 - 0.92 | <0.001 |
| INCP | 0.87 | 0.71 - 1.08 | 0.198 |  | 0.98 | 0.90 - 1.06 | 0.559 |  | 0.96 | 0.89 - 1.04 | 0.287 |
| Residence-hospital distance, miles |  |  |  |  |  |  |  |  |  |  |  |
| ≤10 | 1.00 | - | - |  | 1.00 | - | - |  | 1.00 | - | - |
| (10-20] | 1.11 | 0.94 - 1.30 | 0.211 |  | 0.97 | 0.91 - 1.04 | 0.380 |  | 1.00 | 0.94 - 1.06 | 0.922 |
| (20-50] | 1.17 | 0.99 - 1.38 | 0.060 |  | 0.94 | 0.87 - 1.01 | 0.078 |  | 0.97 | 0.91 - 1.04 | 0.405 |
| >50 | 0.91 | 0.73 - 1.13 | 0.397 |  | 0.93 | 0.85 - 1.02 | 0.142 |  | 0.93 | 0.85 - 1.01 | 0.093 |
| Care transition |  |  |  |  |  |  |  |  |  |  |  |
| None | 1.00 | - | - |  | 1.00 | - | - |  | 1.00 | - | - |
| Yes | 1.02 | 0.90 - 1.16 | 0.709 |  | 0.97 | 0.91 - 1.02 | 0.218 |  | 0.97 | 0.92 - 1.02 | 0.252 |
| **Clinical treatments** |  |  |  |  |  |  |  |  |  |  |  |
| Surgery |  |  |  |  |  |  |  |  |  |  |  |
| Biopsy | - | - | - |  | - | - | - |  | 1.00 | - | - |
| Resection | - | - | - |  | - | - | - |  | 0.87 | 0.82 - 0.93 | <0.001 |
| Adjuvant therapy |  |  |  |  |  |  |  |  |  |  |  |
| Non-CRT | 1.00 | - | - |  | 1.00 | - | - |  | 1.00 | - | - |
| CRT | 0.80 | 0.68 - 0.95 | 0.008 |  | 0.64 | 0.59 - 0.68 | <0.001 |  | 0.66 | 0.62 - 0.71 | <0.001 |
| Charlson/Deyo Score |  |  |  |  |  |  |  |  |  |  |  |
| 0 | 1.00 | - | - |  | 1.00 | - | - |  | 1.00 | - | - |
| 1 | 1.11 | 0.96 - 1.29 | 0.175 |  | 1.19 | 1.12 - 1.27 | <0.001 |  | 1.18 | 1.11 - 1.25 | <0.001 |
| ≥ 2 | 1.17 | 0.98 - 1.39 | 0.080 |  | 1.29 | 1.19 - 1.40 | <0.001 |  | 1.27 | 1.18 - 1.36 | <0.001 |

| Abbreviation: HR, hazard ratio; 95%CI, 95% confidence interval. |
| --- |
| *: Adjusted time to adjuvant therapy (four-category variable), age at diagnosis, gender, period, race/ethnicity, residence, education, income, insurance, facility location, distance, care transition, surgery, adjuvant therapy, and Charlson/Deyo score by using multivariable Cox proportional models in NCDB. |

| **Table S6**. Multivariable Cox models of OS in relation to time to adjuvant therapy from NCDB*. (Two-category) | | | | | | | | | | | |
| --- | --- | --- | --- | --- | --- | --- | --- | --- | --- | --- | --- |
|  | Biopsy (N=1316) | | |  | Resection (N=6845) | | |  | Total (N=8161) | | |
| Predictors | aHR | 95%CI | *P* |  | aHR | 95%CI | *P* |  | aHR | 95%CI | *P* |
| **Time to adjuvant therapy, days** |  |  |  |  |  |  |  |  |  |  |  |
| <26 | 1.00 | - | - |  | 1.00 | - | - |  | 1.00 | - | - |
| ≥27 | 0.84 | 0.74 - 0.95 | 0.006 |  | 0.89 | 0.84 - 0.94 | <0.001 |  | 0.88 | 0.83 - 0.93 | <0.001 |
| **Socio-demographics** |  |  |  |  |  |  |  |  |  |  |  |
| Age at diagnosis, years |  |  |  |  |  |  |  |  |  |  |  |
| 65-74 | 1.00 | - | - |  | 1.00 | - | - |  | 1.00 | - | - |
| 75-90 | 1.50 | 1.32 - 1.71 | <0.001 |  | 1.39 | 1.32 - 1.47 | <0.001 |  | 1.41 | 1.34 - 1.49 | <0.001 |
| Gender |  |  |  |  |  |  |  |  |  |  |  |
| Male | 1.00 | - | - |  | 1.00 | - | - |  | 1.00 | - | - |
| Female | 1.00 | 0.88 - 1.13 | 0.974 |  | 0.96 | 0.91 - 1.01 | 0.103 |  | 0.97 | 0.92 - 1.01 | 0.169 |
| Year of diagnosis |  |  |  |  |  |  |  |  |  |  |  |
| 1/2004-12/2005 | 1.00 | - | - |  | 1.00 | - | - |  | 1.00 | - | - |
| 1/2006-12/2007 | 1.16 | 0.82 - 1.65 | 0.399 |  | 0.96 | 0.88 - 1.05 | 0.403 |  | 0.97 | 0.89 - 1.06 | 0.539 |
| 1/2008-12/2009 | 1.09 | 0.83 - 1.44 | 0.522 |  | 0.98 | 0.90 - 1.06 | 0.602 |  | 0.97 | 0.90 - 1.05 | 0.508 |
| 1/2010-12/2011 | 1.07 | 0.82 - 1.39 | 0.606 |  | 0.90 | 0.83 - 0.98 | 0.015 |  | 0.91 | 0.84 - 0.98 | 0.015 |
| 1/2012-12/2014 | 1.20 | 0.93 - 1.56 | 0.164 |  | 0.88 | 0.81 - 0.95 | 0.001 |  | 0.91 | 0.85 - 0.98 | 0.014 |
| Race/Ethnicity |  |  |  |  |  |  |  |  |  |  |  |
| White | 1.00 | - | - |  | 1.00 | - | - |  | 1.00 | - | - |
| Black | 0.95 | 0.71 - 1.29 | 0.764 |  | 0.89 | 0.78 - 1.02 | 0.106 |  | 0.91 | 0.81 - 1.03 | 0.154 |
| Hispanic | 0.98 | 0.71 - 1.34 | 0.900 |  | 0.76 | 0.66 - 0.88 | <0.001 |  | 0.80 | 0.71 - 0.92 | 0.001 |
| Others | 0.79 | 0.54 - 1.16 | 0.231 |  | 0.76 | 0.63 - 0.91 | 0.003 |  | 0.75 | 0.64 - 0.88 | 0.001 |
| Residential location |  |  |  |  |  |  |  |  |  |  |  |
| Metropolitan | 1.00 | - | - |  | 1.00 | - | - |  | 1.00 | - | - |
| Urban/Rural | 1.06 | 0.88 - 1.28 | 0.510 |  | 1.01 | 0.93 - 1.10 | 0.817 |  | 1.02 | 0.94 - 1.10 | 0.635 |
| Education, % |  |  |  |  |  |  |  |  |  |  |  |
| ≥29 | 1.00 | - | - |  | 1.00 | - | - |  | 1.00 | - | - |
| 20-28.9 | 1.01 | 0.81 - 1.26 | 0.924 |  | 1.13 | 1.03 - 1.25 | 0.013 |  | 1.11 | 1.02 - 1.21 | 0.021 |
| 14-19.9 | 1.04 | 0.83 - 1.31 | 0.747 |  | 1.17 | 1.06 - 1.30 | 0.003 |  | 1.15 | 1.05 - 1.26 | 0.004 |
| <14 | 0.89 | 0.69 - 1.14 | 0.343 |  | 1.19 | 1.07 - 1.33 | 0.002 |  | 1.13 | 1.03 - 1.25 | 0.014 |
| Income, dollars |  |  |  |  |  |  |  |  |  |  |  |
| <30,000 | 1.00 | - | - |  | 1.00 | - | - |  | 1.00 | - | - |
| 30,000-35,999 | 1.10 | 0.87 - 1.39 | 0.425 |  | 0.97 | 0.88 - 1.08 | 0.622 |  | 1.00 | 0.91 - 1.10 | 0.960 |
| 36,000-45,999 | 1.17 | 0.92 - 1.48 | 0.194 |  | 0.92 | 0.82 - 1.02 | 0.107 |  | 0.96 | 0.87 - 1.06 | 0.403 |
| ≥46,000 | 1.07 | 0.82 - 1.40 | 0.611 |  | 0.83 | 0.73 - 0.93 | 0.002 |  | 0.87 | 0.78 - 0.97 | 0.009 |
| Insurance status |  |  |  |  |  |  |  |  |  |  |  |
| Not insured/Medicaid/Other government | 1.00 | - | - |  | 1.00 | - | - |  | 1.00 | - | - |
| Private insurance | 1.57 | 1.04 - 2.37 | 0.030 |  | 0.99 | 0.83 - 1.19 | 0.921 |  | 1.08 | 0.92 - 1.27 | 0.361 |
| Medicare | 1.59 | 1.08 - 2.35 | 0.019 |  | 1.03 | 0.87 - 1.22 | 0.753 |  | 1.12 | 0.95 - 1.31 | 0.169 |
| **Facility characteristics** |  |  |  |  |  |  |  |  |  |  |  |
| Facility location |  |  |  |  |  |  |  |  |  |  |  |
| Northeast | 1.00 | - | - |  | 1.00 | - | - |  | 1.00 | - | - |
| South | 1.22 | 1.03 - 1.44 | 0.020 |  | 1.12 | 1.04 - 1.21 | 0.002 |  | 1.13 | 1.06 - 1.21 | <0.001 |
| Midwest | 1.06 | 0.90 - 1.25 | 0.492 |  | 1.11 | 1.04 - 1.20 | 0.004 |  | 1.10 | 1.03 - 1.18 | 0.004 |
| West | 1.17 | 0.96 - 1.42 | 0.110 |  | 1.04 | 0.96 - 1.14 | 0.326 |  | 1.07 | 0.99 - 1.16 | 0.103 |
| Facility type |  |  |  |  |  |  |  |  |  |  |  |
| Non-AC | 1.00 | - | - |  | 1.00 | - | - |  | 1.00 | - | - |
| AC | 0.80 | 0.71 - 0.92 | 0.001 |  | 0.89 | 0.84 - 0.94 | <0.001 |  | 0.87 | 0.83 - 0.92 | <0.001 |
| INCP | 0.87 | 0.71 - 1.08 | 0.209 |  | 0.97 | 0.90 - 1.06 | 0.523 |  | 0.96 | 0.89 - 1.03 | 0.267 |
| Residence-hospital distance, miles |  |  |  |  |  |  |  |  |  |  |  |
| ≤10 | 1.00 | - | - |  | 1.00 | - | - |  | 1.00 | - | - |
| (10-20] | 1.11 | 0.95 - 1.31 | 0.177 |  | 0.97 | 0.91 - 1.04 | 0.364 |  | 1.00 | 0.94 - 1.06 | 0.903 |
| (20-50] | 1.17 | 1.00 - 1.38 | 0.056 |  | 0.94 | 0.87 - 1.01 | 0.080 |  | 0.97 | 0.91 - 1.04 | 0.417 |
| >50 | 0.92 | 0.74 - 1.13 | 0.419 |  | 0.93 | 0.85 - 1.03 | 0.154 |  | 0.93 | 0.85 - 1.01 | 0.105 |
| Care transition |  |  |  |  |  |  |  |  |  |  |  |
| None | 1.00 | - | - |  | 1.00 | - | - |  | 1.00 | - | - |
| Yes | 1.01 | 0.89 - 1.15 | 0.842 |  | 0.97 | 0.92 - 1.02 | 0.269 |  | 0.97 | 0.92 - 1.02 | 0.264 |
| **Clinical treatments** |  |  |  |  |  |  |  |  |  |  |  |
| Surgery |  |  |  |  |  |  |  |  |  |  |  |
| Biopsy | - | - | - |  | - | - | - |  | 1.00 | - | - |
| Resection | - | - | - |  | - | - | - |  | 0.87 | 0.81 - 0.92 | <0.001 |
| Adjuvant therapy |  |  |  |  |  |  |  |  |  |  |  |
| Non-CRT | 1.00 | - | - |  | 1.00 | - | - |  | 1.00 | - | - |
| CRT | 0.80 | 0.68 - 0.93 | 0.005 |  | 0.63 | 0.59 - 0.68 | <0.001 |  | 0.66 | 0.62 - 0.70 | <0.001 |
| Charlson/Deyo Score |  |  |  |  |  |  |  |  |  |  |  |
| 0 | 1.00 | - | - |  | 1.00 | - | - |  | 1.00 | - | - |
| 1 | 1.11 | 0.96 - 1.28 | 0.177 |  | 1.19 | 1.12 - 1.27 | <0.001 |  | 1.18 | 1.11 - 1.25 | <0.001 |
| ≥ 2 | 1.17 | 0.99 - 1.39 | 0.071 |  | 1.29 | 1.19 - 1.40 | <0.001 |  | 1.27 | 1.18 - 1.36 | <0.001 |

| Abbreviation: HR, hazard ratio; 95%CI, 95% confidence interval. |
| --- |
| *: Adjusted time to adjuvant therapy (two-category variable), age at diagnosis, gender, period, race/ethnicity, residence, education, income, insurance, facility location, distance, care transition, surgery, adjuvant therapy, and Charlson/Deyo score by using multivariable Cox proportional models in NCDB. |

| **Table S7.** Likelihood of delayed timing vs. early timing and the related factors in SEER-Medicare (N=3159)*. | | | | | | | | |
| --- | --- | --- | --- | --- | --- | --- | --- | --- |
|  | Delayed timing vs. Early timing | | |  |  | Delayed timing vs. Early timing | | |
| Predictors | aOR | 95%CI | *P* |  | Predictors | aOR | 95%CI | *P* |
| **Socio-demographics** |  |  |  |  | Marital status |  |  |  |
| Age at diagnosis, years |  |  |  |  | Single/DWS | 1.00 | - | - |
| 65-74 | 1.00 | - | - |  | Married | 0.94 | 0.80 - 1.11 | 0.479 |
| 75-90 | 0.94 | 0.81 - 1.09 | 0.431 |  | Education, % |  |  |  |
| Gender |  |  |  |  | ≥29 | 1.00 | - | - |
| Male | 1.00 | - | - |  | 20-28.9 | 1.03 | 0.78 - 1.37 | 0.818 |
| Female | 1.04 | 0.90 - 1.20 | 0.613 |  | 14-19.9 | 1.01 | 0.75 - 1.37 | 0.929 |
| Year of diagnosis |  |  |  |  | <14 | 0.87 | 0.65 - 1.18 | 0.384 |
| 1/2004-12/2005 | 1.00 | - | - |  | **Facility characteristics** |  |  |  |
| 1/2006-12/2007 | 1.00 | 0.81 - 1.23 | 0.964 |  | Registry location |  |  |  |
| 1/2008-12/2009 | 1.12 | 0.90 - 1.38 | 0.310 |  | Northeast | 1.00 | - | - |
| 1/2010-12/2011 | 0.87 | 0.71 - 1.06 | 0.161 |  | North Central | 1.17 | 0.91 - 1.51 | 0.225 |
| 1/2012-12/2013 | 0.88 | 0.68 - 1.12 | 0.293 |  | South | 1.05 | 0.83 - 1.34 | 0.673 |
| Race/Ethnicity |  |  |  |  | West | 1.14 | 0.94 - 1.38 | 0.193 |
| White | 1.00 | - | - |  | Teaching status |  |  |  |
| Black | 0.95 | 0.64 - 1.42 | 0.818 |  | No | 1.00 | - | - |
| Hispanic | 0.95 | 0.52 - 1.73 | 0.865 |  | Yes | 0.99 | 0.85 - 1.15 | 0.900 |
| Others | 1.30 | 0.88 - 1.94 | 0.192 |  | **Clinical treatments** |  |  |  |
| Residential location |  |  |  |  | Surgery |  |  |  |
| Metropolitan | 1.00 | - | - |  | Biopsy | 1.00 | - | - |
| Urban/Rural | 1.02 | 0.83 - 1.25 | 0.875 |  | STR | 1.31 | 1.10 - 1.56 | 0.002 |
| Income, dollars |  |  |  |  | GTR | 1.41 | 1.19 - 1.67 | 0.000 |
| <30,000 | 1.00 | - | - |  | Charlson Comorbidity Score |  |  |  |
| 30,000-35,999 | 1.05 | 0.77 - 1.44 | 0.750 |  | 0 | 1.00 | - | - |
| 36,000-45,999 | 1.12 | 0.84 - 1.50 | 0.445 |  | 1 | 1.09 | 0.92 - 1.29 | 0.324 |
| ≥46,000 | 1.05 | 0.77 - 1.43 | 0.758 |  | ≥ 2 | 0.85 | 0.71 - 1.03 | 0.090 |
| Abbreviation: OR, odds ratio; 95%CI, 95% confidence interval. *: Adjusted age at diagnosis, gender, period, race/ethnicity, marital status, residence, education, income, registry location, teaching status, surgery, and Charlson Comorbidity Score by using multivariable binary logistic regression model. | | | | | | | | |

| **Table S8.** Likelihood of delayed timing versus early timing and the related factors in NCDB (N=8161).* | | | | | | | | |
| --- | --- | --- | --- | --- | --- | --- | --- | --- |
|  | Delayed timing vs. Early timing | | |  |  | Delayed timing vs. Early timing | | |
| Predictors | aOR | 95%CI | *P* |  | Predictors | aOR | 95%CI | *P* |
| **Socio-demographics** |  |  |  |  | *Table Continued* |  |  |  |
| Age at diagnosis, years |  |  |  |  | Insurance status |  |  |  |
| 65-74 | 1.00 | - | - |  | Not insured/Medicaid/Other government | | |  |
| 75-90 | 0.94 | 0.84 - 1.04 | 0.245 |  | Private insurance | 0.82 | 0.58 - 1.16 | 0.257 |
| Gender |  |  |  |  | Medicare | 0.81 | 0.58 - 1.13 | 0.211 |
| Male | 1.00 | - | - |  | **Facility characteristics** |  |  |  |
| Female | 1.06 | 0.96 - 1.17 | 0.285 |  | Facility location |  |  |  |
| Year of diagnosis |  |  |  |  | Northeast | 1.00 | - | - |
| 1/2004-12/2006 | 1.00 | - | - |  | South | 0.81 | 0.70 - 0.94 | 0.006 |
| 1/2007-12/2008 | 1.20 | 1.00 - 1.43 | 0.044 |  | Midwest | 0.79 | 0.69 - 0.92 | 0.002 |
| 1/2009-12/2010 | 1.74 | 1.47 - 2.06 | <0.001 |  | West | 0.76 | 0.64 - 0.91 | 0.002 |
| 1/2011-12/2012 | 2.16 | 1.84 - 2.53 | <0.001 |  | Facility type |  |  |  |
| 1/2013-12/2014 | 2.39 | 2.05 - 2.78 | <0.001 |  | Non-AC | 1.00 | - | - |
| Race/Ethnicity |  |  |  |  | AC | 1.12 | 1.00 - 1.26 | 0.041 |
| White | 1.00 | - | - |  | INCP | 1.06 | 0.90 - 1.25 | 0.480 |
| Black | 1.38 | 1.04 - 1.82 | 0.023 |  | Residence-hospital distance, miles | |  |  |
| Hispanic | 1.74 | 1.29 - 2.35 | <0.001 |  | 0-10 | 1.00 | - | - |
| Others | 0.80 | 0.58 - 1.11 | 0.188 |  | 10-20 | 1.06 | 0.93 - 1.22 | 0.360 |
| Residential location |  |  |  |  | 20-50 | 1.00 | 0.87 - 1.16 | 0.961 |
| Metropolitan | 1.00 | - | - |  | 50- | 0.84 | 0.70 - 1.00 | 0.056 |
| Urban/Rural | 0.97 | 0.83 - 1.14 | 0.738 |  | Care transition |  |  |  |
| Education, % |  |  |  |  | None | 1.00 | - | - |
| ≥29 | 1.00 | - | - |  | Yes | 1.98 | 1.76 - 2.22 | <0.001 |
| 20-28.9 | 0.96 | 0.79 - 1.16 | 0.657 |  | **Clinical treatments** |  |  |  |
| 14-19.9 | 0.92 | 0.75 - 1.13 | 0.444 |  | Surgery |  |  |  |
| <14 | 0.85 | 0.69 - 1.06 | 0.143 |  | Biopsy | 1.00 | - | - |
| Income, dollars |  |  |  |  | Resection | 1.55 | 1.36 - 1.77 | <0.001 |
| <30,000 | 1.00 | - | - |  | Charlson/Deyo Score |  |  |  |
| 30,000-35,999 | 1.05 | 0.86 - 1.30 | 0.616 |  | 0 | 1.00 | - | - |
| 36,000-45,999 | 1.01 | 0.82 - 1.25 | 0.905 |  | 1 | 1.08 | 0.95 - 1.22 | 0.249 |
| ≥46,000 | 1.05 | 0.84 - 1.33 | 0.651 |  | ≥ 2 | 1.02 | 0.87 - 1.19 | 0.791 |
| Abbreviation: OR, odds ratio; 95%CI, 95% confidence interval; AC, academic center. | | | | | | | | |
| *: Adjusted age at diagnosis, gender, period, race/ethnicity, residential location, education, income, insurance, distance, facility location, facility type, care translon, surgery, and Charlson/Deyo score by using multivariable binary logistic regression models. | | | | | | | | |

| **Table S9**. Multivariable Cox models of OS for the PSM matched cohort from SEER-Medicare and NCDB. | | | | | |
| --- | --- | --- | --- | --- | --- |
|  | Matched sub-sample^*^ | | | | |
| Predictors | aHR | 95%CI | *P* | LR | AIC |
| ***SEER-Medicare, N=3048^a^*** |  |  |  |  |  |
| **Time to adjuvant therapy, days** |  |  |  |  |  |
| Univariable analysis |  |  |  |  |  |
| Delayed timing vs. Early timing | 0.87 | 0.81 - 0.93 | <0.001 | -33074.93 | 66151.86 |
| Adjust all covariates |  |  |  |  |  |
| Delayed timing vs. Early timing | 0.88 | 0.82 - 0.95 | 0.001 | -20535.26 | 41124.53 |
| Adjust all covariates+propensity score |  |  |  |  |  |
| Delayed timing vs. Early timing | 0.88 | 0.82 - 0.95 | 0.001 | -20534.88 | 41125.77 |
|  |  |  |  |  |  |
| ***NCDB, N=4776^b^*** |  |  |  |  |  |
| **Time to adjuvant therapy, days** |  |  |  |  |  |
| Univariable analysis |  |  |  |  |  |
| Delayed timing vs. Early timing | 0.84 | 0.79 - 0.89 | <0.001 | -33074.93 | 66151.86 |
| Adjust all covariates |  |  |  |  |  |
| Delayed timing vs. Early timing | 0.89 | 0.84 - 0.95 | <0.001 | -32857.68 | 65779.35 |
| Adjust all covariates+propensity score |  |  |  |  |  |
| Delayed timing vs. Early timing | 0.89 | 0.84 - 0.95 | <0.001 | -32857.48 | 65780.95 |
| Abbreviation: HR, hazard ratio; 95%CI, 95% confidence interval; LR, likelihood ratio; AIC, Akaike information criterion. *: Adjusted time to adjuvant therapy, age at diagnosis, gender, period, race/ethnicity, marital status, residence, education, income, registry location, surgery, and Charlson Comorbidity Score based on PSM matched sub-sample for SEER-Medicare.   Adjusted time to adjuvant therapy, age at diagnosis, gender, period, race/ethnicity, residence, education, income, insurance, facility location, distance, care transition, surgery, adjuvant therapy, and Charlson/Deyo score based on PSM matched sub-sample for NCDB. | | | | | |
